# Targeting the YAP/TAZ Pathway in Uveal and Conjunctival Melanoma With Verteporfin

**DOI:** 10.1167/iovs.62.4.3

**Published:** 2021-04-02

**Authors:** Niels J. Brouwer, Eleni K. Konstantinou, Evangelos S. Gragoudas, Marina Marinkovic, Gregorius P. M. Luyten, Ivana K. Kim, Martine J. Jager, Demetrios G. Vavvas

**Affiliations:** 1 Department of Ophthalmology, Retina Service, Angiogenesis Laboratory, Massachusetts Eye and Ear Infirmary, Harvard Medical School, Boston, MA, United States; 2 Department of Ophthalmology, Leiden University Medical Center, Leiden, The Netherlands

**Keywords:** eye disease, oncology, ocular melanoma, uveal melanoma, YAP1, verteporfin, cell lines

## Abstract

**Purpose:**

The purpose of this study was to determine whether YAP/TAZ activation in uveal melanoma (UM) and the susceptibility of melanoma cell lines to YAP/TAZ inhibition by verteporfin (VP) is related to the tumor's genetic background.

**Methods:**

Characteristics of 144 patients with enucleated UM were analyzed together with mRNA expression levels of YAP/TAZ-related genes (80 patients from the The Cancer Genome Atlas [TCGA] project and 64 patients from Leiden, The Netherlands). VP was administered to cell lines 92.1, OMM1, Mel270, XMP46, and MM28 (UM), CRMM1 and CRMM2 (conjunctival melanoma), and OCM3 (cutaneous melanoma). Viability, growth speed, and expression of YAP1-related proteins were assessed.

**Results:**

In TCGA data, high expression of *YAP1* and *WWTR1* correlated with the presence of monosomy 3 (*P* = 0.009 and *P* < 0.001, respectively) and BAP1-loss (*P* = 0.003 and *P* = 0.001, respectively) in the primary UM; metastasis development correlated with higher expression of *YAP1* (*P* = 0.05) and *WWTR1* (*P* = 0.003). In Leiden data, downstream transcription factor *TEAD4* was increased in cases with M3/BAP1-loss (*P* = 0.002 and *P* = 0.006) and related to metastasis (*P* = 0.004). UM cell lines 92.1, OMM1, and Mel270 (*GNAQ/11-*mutation, BAP1-positive) and the fast-growing cell line OCM3 (*BRAF-*mutation) showed decreased proliferation after exposure to VP. Two slow-growing UM cell lines XMP46 and MM28 (*GNAQ/11*-mutation, BAP1-negative) were not sensitive to VP, and neither were the two conjunctival melanoma cell lines (*BRAF/NRAS-*mutation).

**Conclusions:**

High risk UM showed an increased expression of YAP/TAZ-related genes. Although most UM cell lines responded in vitro to VP, BAP1-negative and conjunctival melanoma cell lines did not. Not only the mutational background, but also cell growth rate is an important predictor of response to YAP/TAZ inhibition by VP.

Uveal melanoma (UM) is the most common primary intraocular malignant tumor in adults, with an incidence of approximately 5 to 6 per million in the United States.^1^^,^^2^ Treatment includes various forms of radiotherapy, removal of the eye is a last resort option.^3^^–^^5^ Up to 50% of patients develop metastasis,^6^ and no proper treatment for metastatic disease is as yet available.^7^

Conjunctival melanoma (CoM) is rarer than UM, with an incidence of approximately 0.7 per million in Caucasians.^8^^–^^10^ Treatment consists generally of excision and adjuvant therapy (e.g. radiotherapy or topical chemotherapy)^11^; even so, 7% to 32% of patients die from metastases.^12^^–^^14^

Although both lesions are related to the eye, the genetic background of UM and CoM differs. UM is known to have driver mutations in *GNAQ/11*,^15^^,^^16^ *CYSLTR2*,^17^ and *PLCB4*,^18^ with subsequent mutations in *BAP1* (associated with adverse prognosis), *SF3B1* (associated with late metastasis), or *EIF1AX* (associated with good prognosis).^19^ CoM on the other hand resembles cutaneous melanoma and has driver mutations in *BRAF*, *NRAS*, *Kit*, *TERT*, or *NF1.*^20^^–^^25^ Despite their different backgrounds, UM and CoM share the need for the development of new and effective therapies.^26^

Recent studies identified the importance of the YAP/TAZ pathway in oncology, for tumor growth and possible targeting.^27^ The YAP/TAZ pathway is involved in normal cell proliferation and apoptosis, regulating organ size. Key components of this pathway are Yes-Associated Protein 1 (YAP1) and its co-activator TAZ (a.k.a. WWTR1, not to be confused with the unrelated *Tafazzin* gene). YAP1 and TAZ can bind to TEAD proteins in the cell nucleus, allowing them to read DNA, and activate several genes that promote cell growth and proliferation (e.g. *CTGF*, *CYR61*, and *Survivin*).^28^ In various cancers, including cutaneous melanoma, increased activity of the YAP/TAZ pathway has been related to worse survival,^29^^,^^30^ and inhibition of YAP/TAZ has been suggested as a potential new therapy.^27^ Interestingly, the YAP/TAZ pathway can be blocked pharmacologically, using the benzoporphyrin verteporfin (VP, trade name: Visudyne). VP is being used clinically as a photosensitizer in photodynamic therapy (PDT) for various retinal disorders.^31^ In PDT, upon irradiation with a nonthermal laser, reactive oxygen species are formed causing damage to the endothelium and regression of vessels. VP blocks YAP/TAZ through a different mechanism, however, as it can disrupt the YAP-TEAD complexes even without light activation.^32^ Via this mechanism, VP inhibited in vitro cell growth in several cancers, such as retinoblastoma^33^ and glioma.^34^

Approximately 90% of UM harbor a *GNAQ/11* mutation,^15^^,^^16^^,^^19^ which was found to activate the YAP/TAZ cascade.^35^^,^^36^ Inhibition of this pathway by shRNA or drugs led to decreased cell growth in vitro as well as tumor regression in mouse models carrying a *GNAQ/11* mutation.^35^^,^^36^ This leads to the question whether the YAP/TAZ pathway can be used as a therapeutic target in UM. The *GNAQ/11* mutation is absent in CoM,^16^^,^^37^ but other stimuli (such as mechanical stress and receptor signaling) can activate the YAP/TAZ cascade as well.^38^ YAP1 expression was detected in cutaneous melanoma cell lines lacking a *GNAQ/11* mutation (but harboring *BRAF* or *NRAS* mutations instead),^35^^,^^39^^,^^40^ and in human cutaneous melanoma tissue where a high expression was related to worse survival.^29^^,^^30^ Results of YAP/TAZ inhibition in cutaneous melanoma are mixed: one study identified diminished cell growth in cutaneous melanoma cell lines after administration of VP but found no effect on tumor development or tumor growth in a mouse model,^39^ whereas another study found no effect of YAP/TAZ inhibition using shRNA on in vitro proliferation, but identified decreased in vitro invasiveness and less metastases formation after injection of melanoma cells in mice.^40^ To our knowledge, no studies exist on YAP/TAZ inhibition in CoM.

Recently, it was reported that the YAP/TAZ pathway has little prognostic value for patient survival in UM.^41^ This mechanism is poorly understood, however, and it is unknown if the YAP/TAZ pathway (activated by the early *GNAQ/11* mutation) is altered by chromosome changes or other mutations, such as in *BAP1*, which is known to be related to adverse prognosis.^42^^,^^43^ Interestingly, the genes coding for *BAP1* as well as *TAZ* are located on chromosome 3. Hypothesizing a link between the genetic make-up of UM and YAP/TAZ activity, we wondered if UM cells lacking BAP1 expression are more susceptible to treatment with VP, and whether CoM cells are sensitive at all.

We set out to investigate whether mRNA expression of YAP1-related genes was related to clinical, histological, and genetic tumor characteristics in UM. Next, we studied the effect of YAP1-inhibition using VP without light activation on multiple UM cell lines with different genetic profiles (including cell lines with and without BAP1 expression), and included CoM cell lines with either a *BRAF* or *NRAS* mutation as a control. We show that the YAP/TAZ pathway has a higher activity in UM tissue with unfavorable genetic characteristics such as monosomy 3 (M3)/BAP1 loss. We confirm that VP inhibits growth of BAP1-positive UM cells in vitro, whereas it has limited effect on BAP1-negative cells and CoM, and observed that not only the genetic background, but other traits, such as cell growth rate, were major determinants of VP response.

## Methods

### Patient and Tumor Data

Data from two independent sets of patients with UM were analyzed. The first set was comprised of 80 patients with UM from The Cancer Genome Atlas (TCGA) project (http://cancergenome.nih.gov/). The second set was comprised of 64 patients with UM who underwent primary enucleation at the Leiden University Medical Center (The Netherlands).

From the TCGA project, data on mRNA expression were retrieved for 80 cases.^44^ In this set, the median follow-up time was 26.0 months. BAP1 expression was provided as mRNA expression levels, and dichotomized at the median into BAP1-positive and BAP1-negative cases.^45^

All Leiden patients had been treated by primary enucleation between 1999 and 2008. Clinical and survival data were retrieved from patient medical files, and complemented with data from the Dutch national cancer registry (Registratie Applicatie Nederlandse Kankerregistratie [RANK]).

Messenger RNA was isolated from frozen tumor material for gene expression analysis using the RNeasy Mini Kit (Qiagen, Venlo, The Netherlands). The Illumina HT-12 version 4 chip was used to determine gene expression levels (Illumina, San Diego, CA, USA).

DNA was isolated for single-nucleotide polymorphism (SNP) analysis using the QIAmp DNA Mini kit (Qiagen, Venlo, The Netherlands). With the Affymetrix 250K_NSP microarray and Affymetrix Cytoscan HD chip (Affymetrix, Santa Clara, CA, USA), status of chromosome 3 was determined.^46^ Status of chromosome 8q was additionally identified with digital polymerase chain reaction (dPCR).^46^ BAP1 expression status was assessed by an experienced ocular pathologist with immunohistochemistry (IHC) as previously described^47^ and categorized as BAP1-positive or BAP1-negative. Further details on the determination of chromosome 3/8q status, and IHC of BAP1 were described before.^48^^,^^49^

The study was approved by the Biobank Committee of the Leiden University Medical Center (LUMC; 19.062.CBO/uveamelanoomlab-2019-3; B20.023). The tenets of the Declaration of Helsinki were followed.

### Cell Lines and Culturing

Human uveal melanoma cell lines 92.1 (BAP1*-*pos, *GNAQ*-mut),^50^ OMM1 (BAP1-pos, *GNA11*-mut),^51^ Mel270 (BAP1-pos, *GNAQ*-mut),^52^ XMP46 (BAP1-neg, *GNAQ*-mut),^53^ MM28 (BAP1-neg, *GNA11*-mut),^53^ human conjunctival melanoma cell lines CRMM1 (*BRAF*-mut),^54^ CRMM2 (*NRAS*-mut),^54^ and human melanoma cell line OCM3 (BAP1-pos, *BRAF*-mut)^55^ were studied. An overview of studied cell lines and their genetic mutations is provided in Supplementary Table S1.^56^

Cell lines 92.1, OCM3, OMM1, and Mel270 were grown in RPMI 1640 medium (Gibco, Life Technologies Co.) supplemented with 10% fetal bovine serum (FBS; Gibco, Life Technologies Co.) and 1% antibiotics (10,000 units/mL Penicillin, 10,000 ug/mL Streptomycin; Gibco, Life Technologies Co.). Cell lines XMP46 and MM28 were grown in IMDM medium (Sigma-Aldrich, UK) supplemented with 20% FBS and 2% antibiotics. Cell lines CRMM1 and CRMM2 were grown in F-12K medium (Gibco, Life Technologies Co.) supplemented with 10% FBS and 1% antibiotics. Cells were incubated in a humidified atmosphere of 5% CO2 at 37°C. Cells were protected from light using aluminum foil, and the experiments were performed under dimmed lights.

### Investigated Drugs

The investigated drug was liposomal verteporfin in phosphate buffered saline (PBS; original VP dilution 2 mg/mL; Novartis AG, distributed by Valeant Ophthalmics, Bridgewater, NJ, USA). As a control, PBS (Gibco, Life Technologies Co., Grand Island, NY, USA) was used. Drugs or controls were added to regular cell culture medium of the respective cell lines, in concentrations as described with the experimental designs.

### Viability Assays

Cell viability was assessed using the Cell Counting Kit-8 (Dojindo Molecular Technologies, Rockville, MD, USA). In this assay, a tetrazolium salt (WST-8) is reduced by dehydrogenase activity into a yellow/orange formazan dye. Light absorbance thereby reflects the activity of living cells. Cells were seeded in a 96-well plate at a density of 10,000 cells per well. The following day, various concentrations of VP were added. After 3 days, all wells were gently washed with fresh medium (to remove staining from VP) and the WST-8 salt was added according to the manufacturer's guideline. Light absorbance at 450 nm was measured using a microplate reader and normalized to control values. Experiments were performed in triplicate.

### Growth Curves

Cells were seeded in 6-well plates at a density of 300,000 cells per well. The following day, culture medium was replaced by new medium with the addition of 1.25 ug/mL VP, 7.5 ug/mL VP or PBS. At days 2, 4, and 6, cell numbers were determined using the trypan blue (0.4%) dye exclusion method in an automated cell counter (Invitrogen, Countess II FL). Culture medium (with drugs or control, as mentioned previously) was refreshed on days 2 and 4 for the remaining wells. Experiments were performed in triplicate.

### Protein Expression

Cells were seeded in 6-well plates at a density of 800,000 cells per well. The following day, culture medium was replaced by new medium with the addition of 1.25 ug/mL VP, 7.5 ug/mL VP or PBS. After 24-hour incubation, cells were washed with ice-cold PBS and lysed with MPER with a protease and phosphatase inhibitor. Samples were sonicated for 15 seconds, and centrifugated for 20 minutes at 14,000 g in a precooled 4°C centrifuge. The supernatant was used for further analyses.

Per lane, 20 ug of protein were loaded on a 4% to 12% Bis-Tris gel (NuPage, Invitrogen). After electrophoresis, the assay was transferred to a polyvinylidene difluoride (PVDF) membrane (Millipore, Billerica, MA, USA). Coomassie blue staining was used to ensure equal loading. The membrane was blocked for 1 hour at room temperature in 5% milk and incubated for 3 hours with the respective primary antibodies at a 1:1000 dilution. After washing, the membrane was incubated for 1.5 hours with the respective secondary antibodies at a 1:2000 dilution. Protein expression was visualized with the ECL technique (Amersham ECL Select). Antibodies were purchased from Cell Signaling Technology (Danvers, MA, USA): YAP (4912S), TEAD1 (12292S), and c-Myc (9402S).

### Statistics

Data were analyzed using SPSS version 23. The applied statistical tests were the Mann-Whitney *U* test (numerical parameters, 2 groups) or the Jonckheere test for trends (numerical parameters, more than 2 groups). The Spearman's rho was applied for analysis of correlations. Survival was analyzed with the Kaplan-Meier method and log-rank tests. When applicable, “high” and “low” expression of mRNA values was categorized based on the median expression values. Two-sided tests were reported, and *P* values < 0.05 were considered statistically significant.

## Results

### The YAP/TAZ Pathway is Related to Tumor Characteristics in UM

To study the activation of the YAP/TAZ pathway in human UM, we first analyzed the mRNA expression of YAP1-related genes in UM samples in two independent datasets. One set was comprised of material from 80 UM from the TCGA project, the other set of 64 UM from patients who underwent an enucleation in the LUMC (The Netherlands). In the TCGA dataset, probes were available for *YAP1*, *WWTR1* (=*TAZ*), and *TEAD1*. In the Leiden dataset, probes were available for *YAP1* and *TEAD4*, but not for the other YAP1-related genes.

Both in the TCGA and Leiden datasets, expression of YAP1-related genes did not vary based on patient age, American Joint Commission on Cancer (AJCC) stage, or tumor prominence (Tables 1, 2). In the TCGA dataset, increased *WWTR1* was associated with a greater largest basal diameter (LBD; Spearman correlation 0.323, *P* = 0.004) and a mixed/epithelioid cell type (*P* = 0.002). Interestingly, a higher expression of YAP1 was noticed for lightly pigmented tumors in both data sets compared to highly-pigmented cases (TCGA: *P* = 0.006 and Leiden: *P* = 0.007).

**Table 1.** Clinical Characteristics of the TCGA Study Group and mRNA Expression Levels of YAP1, WWTR1, and TEAD1

	**Total**						
	***N* = 80**	**YAP1**		**WWTR1**		**TEAD1**	
**Categorical**	Cases, %	Median	*P* Value	Median	*P* Value	Median	*P* Value
Gender							
M	45	11.0	0.659	6.8	0.652	10.6	0.476
F	35	11.0		6.8		10.7	
TNM cat, 8th							
T1	0	NA	0.407	NA	0.092	NA	0.115
T2	14	10.9		5.8		10.8	
T3	32	10.9		6.8		10.7	
T4	34	11.1		7.0		10.6	
Pigmentation							
Light	39	11.1	0.006	6.6	0.099	10.9	<0.001
Dark	41	10.7		7.1		10.4	
Cell type							
Spindle	43	10.9	0.092	6.1	0.002	10.7	0.904
Mixed + epithelioid	37	11.1		7.2		10.7	
Ciliary body involvement							
No	64	10.9	0.234	6.6	0.243	10.6	0.012
Yes	16	11.0		7.1		10.9	
Metastasis							
No	53	10.8	0.050	6.5	0.006	10.7	0.552
Yes	27	11.2		7.4		10.7	
Mel.-related death							
No	60	10.9	0.117	6.5	0.003	10.7	0.437
Yes	20	11.2		7.4		10.6	
Necrosis							
No	63	11.0	0.568	6.6	0.256	10.7	0.381
Yes	17	10.9		7.0		10.5	
GNAQ/11 or WT							
No mutation, both WT	6	10.9	0.574	6.95	0.285	10.78	0.139
Any GNAQ/11 mutation	72	11.0		6.65		10.65	
GNAQ or GNA11 status^*^							
GNAQ-mutation	38	11.0	0.752	10.6	0.030	10.6	0.701
GNA11-mutation	34	11.0		10.7		10.7	
	**Total**	**Correlation**		**Correlation**		**Correlation**	
**Numerical**	***N* = 80**	**Spearman**	***P* Value**	**Spearman**	***P* Value**	**Spearman**	***P* Value**

Age – median	61.5	0.007	0.953	0.032	0.778	−0.138	0.221
LBD – median	16.0	0.085	0.461	0.323	0.004	−0.079	0.491
Prominence – median	11.0	0.154	0.185	0.078	0.501	−0.002	0.985

The mRNA expression concerns the individual intensity of each gene.

LBD, largest basal diameter; Mel., melanoma; NA, not applicable; WT, wild type.

* Includes mutually exclusive cases only. In 6 cases, no *GNAQ* or *GNA11* mutation was found; in 2 cases, both *GNAQ* and *GNA11* were mutated.

**Table 2.** Clinical Characteristics of the Leiden Study Group and mRNA Expression Levels of YAP1 and TEAD4

	**Total**				
	***N* = 64**	**YAP1**		**TEAD4**	
**Categorical**	Cases (%)	Median	*P* Value	Median	*P* Value
Gender					
M	33	8.3	0.234	8.0	0.043
F	31	8.4		8.3	
TNM cat, 8th					
T1	6	8.3	0.173	8.1	0.100
T2	25	8.4		8.0	
T3	31	8.3		8.1	
T4	2	8.1		8.1	
Pigmentation					
Light	43	8.4	0.007	8.2	0.469
Dark	20	8.2		8.0	
Cell type					
Spindle	22	8.3	0.932	8.1	0.745
Mixed + epithelioid	42	8.3		8.1	
Ciliary body involvement					
No	40	8.4	0.031	8.0	0.230
Yes	23	8.3		8.2	
Metastasis					
No	27	8.4	0.305	8.0	0.004
Yes	37	8.3		8.2	
Mel.-related death					
No	27	8.4	0.305	8.0	0.004
Yes	37	8.3		8.2	
Necrosis					
No	38	8.2	0.008	8.0	0.318
Yes	26	8.5		8.2	
GNAQ/11 or WT					
No mutation, both WT	4	8.6	0.033	8.0	0.841
Any GNAQ/11 mutation	60	8.3		8.1	
GNAQ or GNA11 status^*^					
GNAQ-mutation	28	8.3	0.468	8.1	0.424
GNA11-mutation	32	8.3		8.1	
	**Total**	**Correlate**		**Correlate**	
**Numerical**	***N* = 64**	**Spearman**	***P* Value**	**Spearman**	***P* Value**

Age – median	61.6	0.040	0.751	0.052	0.684
LBD – median	13.0	−0.148	0.244	0.203	0.107
Prominence – median	8.0	−0.171	0.176	0.178	0.160

The mRNA expression concerns the individual intensity of each gene.

LBD, largest basal diameter; Mel., melanoma; NA, not applicable; WT, wild type.

* Includes mutually exclusive cases only. In 4 cases, no *GNAQ* or *GNA11* mutation was found.

### YAP1-Related Genes are Associated With Unfavorable Tumor Genetics

As the YAP/TAZ pathway is activated by mutations in *GNAQ/11*, we examined the expression of mRNA in tumors with and without these mutations. In the TCGA dataset, tumors with either a *GNAQ* or *GNA11* mutation (*n* = 72) did not differ in their expression of YAP1-related genes compared with tumors without these mutations (*n* = 6; see Table 1). In the Leiden dataset, the four tumors that lacked a GNAQ/11 mutation had a higher YAP1 expression, but a similar TEAD4 expression, than the tumors with a *GNAQ/11* mutation (*n* = 60, *P* = 0.033 and *P* = 0.84, respectively; see Table 2); the interpretation of this finding is hampered, however, due to low numbers of cases lacking a *GNAQ/11-*mutation.

We then tested whether YAP1 activity relates to the genetic status of UM, such as monosomy 3 (M3)/BAP1-loss, or gain of chromosome 8q, two adverse prognostic factors. In the TCGA dataset, both M3 and BAP1-loss were associated with a higher expression of *YAP1* (*P* = 0.009 and *P* = 0.003, respectively) and *WWTR1* (*P* < 0.001 and *P* = 0.001, respectively; Table 3). Although *YAP1* did not differ between M3/BAP1-loss and D3/BAP1-positive UM in the Leiden data, *TEAD4* was expressed higher in M3/BAP1-loss cases (*P* = 0.002 and *P* = 0.006, respectively). Gain of chromosome 8q related to a higher expression of *WWTR1* in the TCGA data (*P* < 0.001) but a lower expression of *TEAD1* (*P* = 0.025), whereas no association with 8q status were observed in the Leiden data. From these data, we conclude that the chromosome 3/BAP1 status of UM is related to the expression of YAP1-related genes, with a higher activity in the prognostically unfavorable cases.

**Table 3.** The mRNA Expression of YAP1-Related Genes Related to Status of Chromosome 3 and 8q, and BAP1 Protein

	**TCGA Data**								**Leiden Data**				
	***N* = 80**	**YAP1**		**WWTR1**		**TEAD1**			***N* = 54**	**YAP1**		**TEAD4**	
	Cases (%)	Median	*P* Value	Median	*P* Value	Median	*P* Value		Cases (%)	Median	*P* Value	Median	*P* Value
Chromosome 3													
Disomy	37 (46)	10.7	0.009	5.7	<0.001	10.8	0.020		20	8.4	0.986	7.9	0.002
Monosomy	37 (46)	11.1		7.4		10.5			34	8.4		8.2	
Chromosome 8q													
Normal	21 (26)	11.1	0.403	5.2	<0.001	10.9	0.025		13	8.4	0.326	8.0	0.213
Gain	59 (74)	10.9		7.2		10.6			41	8.3		8.1	
BAP1 expression													
Negative	40 (50)	11.1	0.003	7.3	0.001	10.6	0.191		30	8.4	0.169	8.2	0.006
Positive	40 (50)	10.7		6.0		10.8			24	8.3		7.9	

The mRNA expression concerns the individual intensity of each gene.

### YAP1-Related Genes are Modestly Associated With Worse Clinical Outcome in UM

In the TCGA dataset, patients who developed metastasis had a higher expression of *WWTR1* (*P* = 0.003) and a borderline insignificant higher expression of *YAP1* (*P* = 0.050) compared to patients without metastases (median follow-up time 26 months; see Table 1). In the Leiden data, *YAP1* was not related to the development of metastases (*P* = 0.31) but a higher expression of *TEAD4* was (*P* = 0.004, median follow-up time 62 months; see Table 2). These findings indicate that high activity of (components of) the YAP/TAZ pathway is modestly associated with a worse clinical outcome in UM.

### VP Inhibits Cell Growth in a Dose-Dependent Manner in Cell Lines With a GNAQ/11 Mutation, But Not in Cell Lines With a BRAF/NRAS Mutation

**Figure 1.**
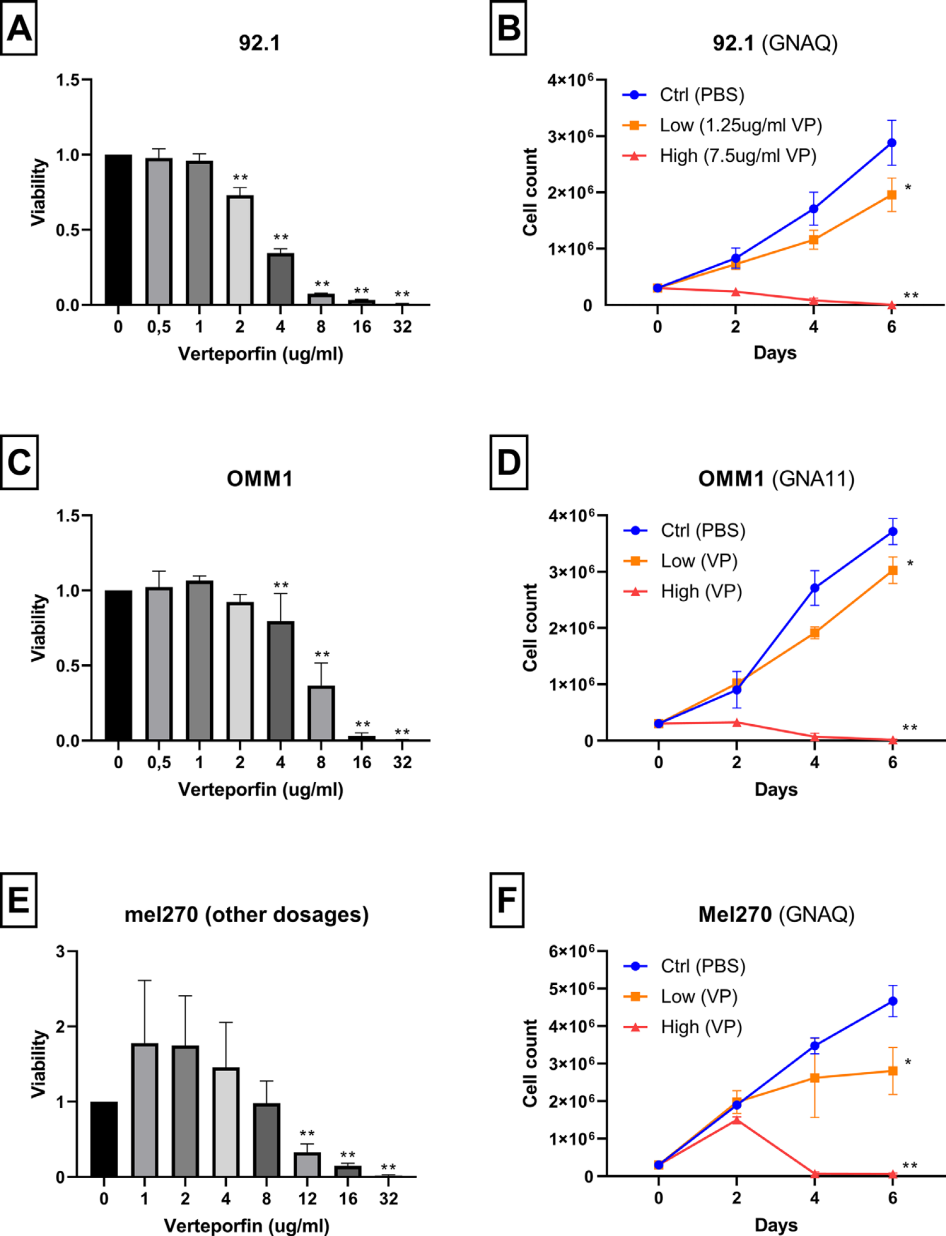
Viability and cell growth after verteporfin treatment. (**A, B**) UM cell line 92.1: *GNAQ*-mutation, BAP1-positive. (**C, D**) UM cell line OMM1: *GNA11*-mutation, BAP1-positive. (**E, F**) UM cell line Mel270: *GNAQ*-mutation, BAP1-positive. Values show mean ± SD of three experiments. In **A**, **C**, and **E**, measurements at each concentration of VP were compared to 0 ug/mL; in **B**, **D**, and **E**, cell counts at the final day were compared between control and low, and between control and high concentrations. The *P* values are indicated by * (*P* < 0.05) or ** (*P* < 0.01).

Next, we studied the effect of YAP/TAZ inhibition in UM and CoM cell lines using VP without light activation. First, we analyzed the inhibitory effect of VP treatment on BAP1-positive UM cell lines with a mutation in *GNAQ* or *GNA11*. Following 3 days of incubation with VP, UM cell lines 92.1 (*GNAQ-mut*), OMM1 (*GNA11-mut*), and Mel270 (*GNAQ-mut*) demonstrated more cell death with increased dosages of VP (Figs. 1A, 1C, 1E). When cultured for a total of 6 days, a low dose of VP was noticed to have only a minor effect on cell growth, whereas a high dose caused complete inhibition (Figs. 1B, 1D, 1F).

**Figure 2.**
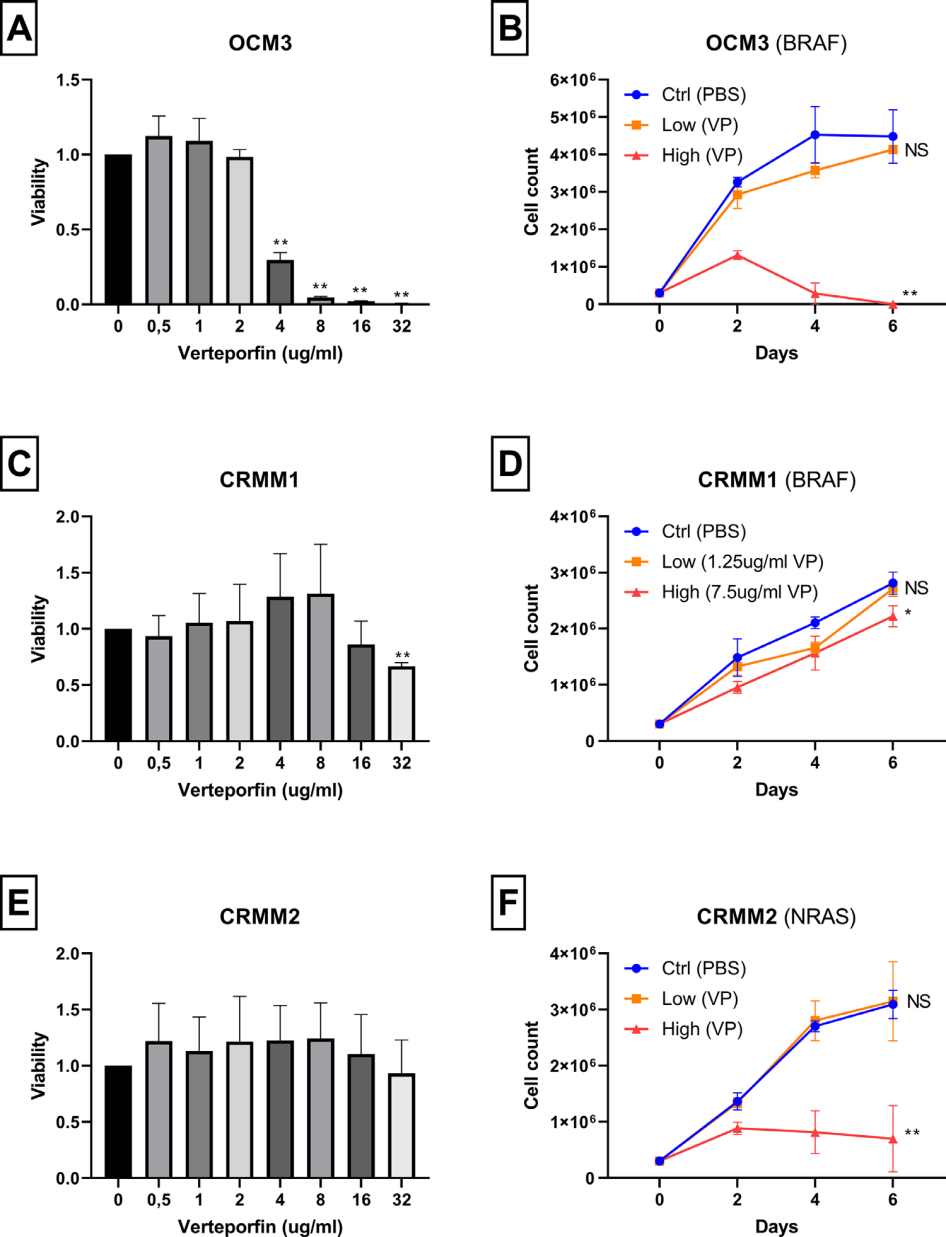
Viability and cell growth after verteporfin treatment. (**A, B**) Melanoma cell line OCM3: *BRAF*-mutation. (**C, D**) CoM cell line CRMM1: *BRAF*-mutation. (**E, F**) CoM cell line CRMM2: *NRAS*-mutation. Values show mean ± SD of three experiments. In **A**, **C**, and **E**, measurements at each concentration of VP were compared to 0 ug/mL; in **B**, **D**, and **E**, cell counts at the final day were compared between control and low, and between control and high concentrations. The *P* values are indicated by * (*P* < 0.05), ** (*P* < 0.01), or not significant (NS) (*P* > 0.05).

We compared the results in the UM cell lines with the effect on cell lines with a *BRAF* or *NRAS* mutation (i.e. melanoma cell line OCM3 [*BRAF-mut*] and the CoM cell lines CRMM1 [*BRAF-mut*] and CRMM2 [*NRAS-mut*]). Cell line OCM3 was sensitive to VP treatment at higher doses, with a remarkable drop in cell viability after treatment for 3 days with >2 ug/mL (Fig. 2A). This could point at nonspecific toxicity of VP rather than a specific effect due to YAP1 inhibition. Both CoM cell lines were not sensitive to VP even at high doses, showing unaltered cell viability (Figs. 2C, 2E). Although the growth curves of CRMM1 and CRMM2 demonstrate a reduced growth speed with high dose VP administration, cell counts were not reduced to zero (Figs. 2D, 2F).

### UM Cell Lines Lacking BAP1 Expression are Not Sensitive to Treatment With VP

As we had noticed that UM tissues with M3/BAP1-loss show a higher mRNA expression of actors in the YAP1 pathway, we now compared the susceptibility of BAP1-expressing and BAP1-negative UM cell lines to VP.

**Figure 3.**
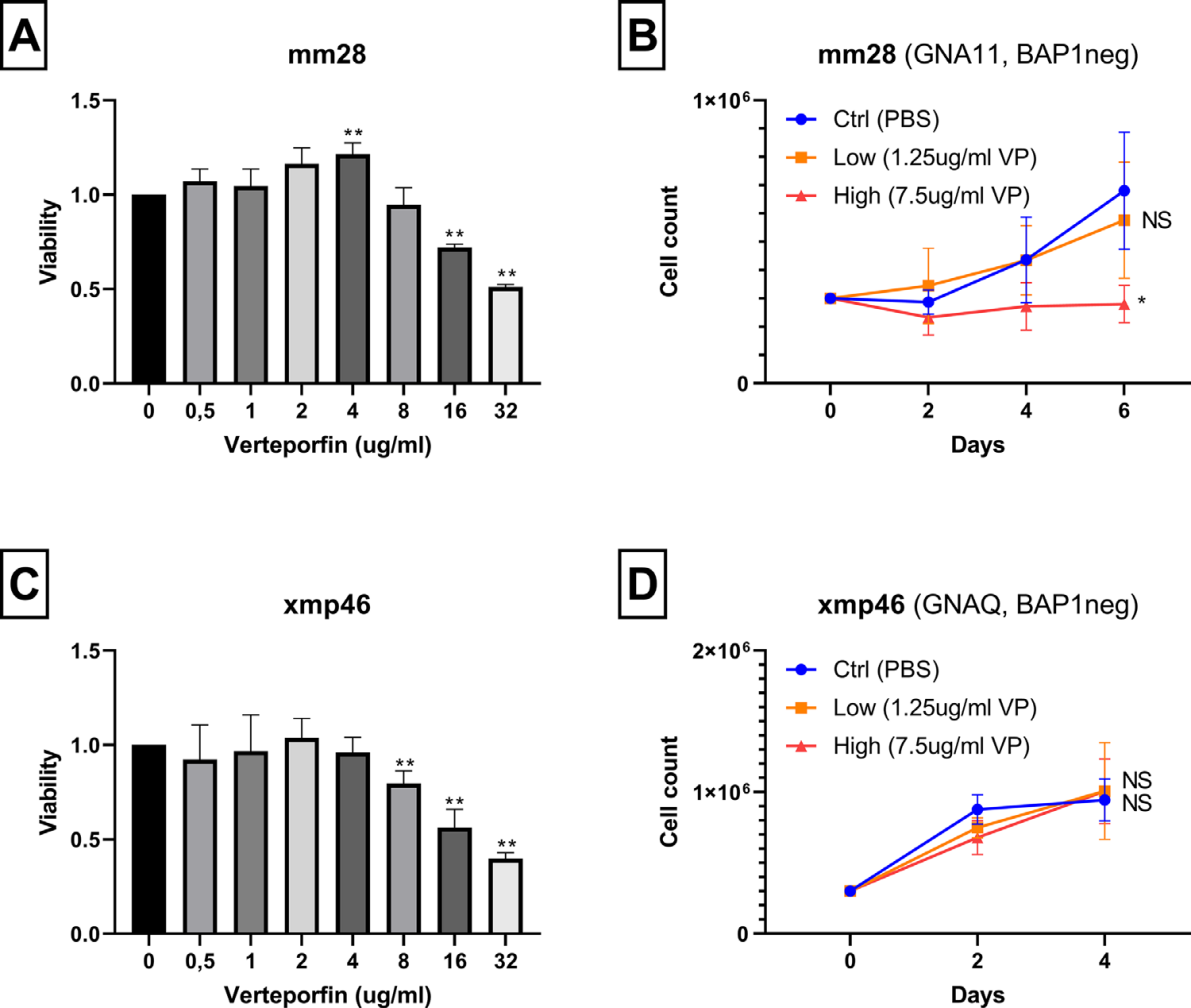
Viability and cell growth after verteporfin treatment. (**A, B**) UM cell line mm28: *GNA11*-mutation, BAP1-negative. (**C, D**) UM Cell line xmp46: *GNAQ*-mutation, BAP1-negative. Values show mean ± SD of three experiments. In **A** and **C**, measurements at each concentration of VP were compared to 0 ug/mL; in **B** and **D**, cell counts at the final day were compared between control and low, and between control and high concentrations. The *P* values are indicated by * (*P* < 0.05), ** (*P* < 0.01) or not significant (NS) (*P* > 0.05).

We included two recently developed UM cell lines with a *GNAQ/11* mutation, which lack expression of BAP1 (i.e. cell line MM28 [*GNA11-mut*, BAP1-neg] and cell line XMP46 [*GNAQ-mut*, BAP1-neg]). Viability assays demonstrated a relative tolerance for VP at low dosages, whereas a dose-dependent decrease of viability tended to occur in both cell lines at dosages >4 ug/mL VP (Figs. 3A, 3C), however, with a smaller effect than in the BAP1-positive UM cell lines.

Remarkably, cell growth experiments demonstrated that the cell numbers of the BAP1-negative cell lines were little affected by either low or high VP concentrations. It should be noticed, however, that these cell lines grew at a much slower rate than the other lines (Figs. 3B, 3D). As the YAP1 pathway is involved in growth, an absence of robust growth may cause insensitivity to YAP1 inhibition. To our knowledge, no fast-growing BAP1-negative UM cell lines exist.

### Not Only the Genetic Background, But Also Cell Growth Rate Predicts Susceptibility for VP of the Various Cell Lines

**Figure 4.**
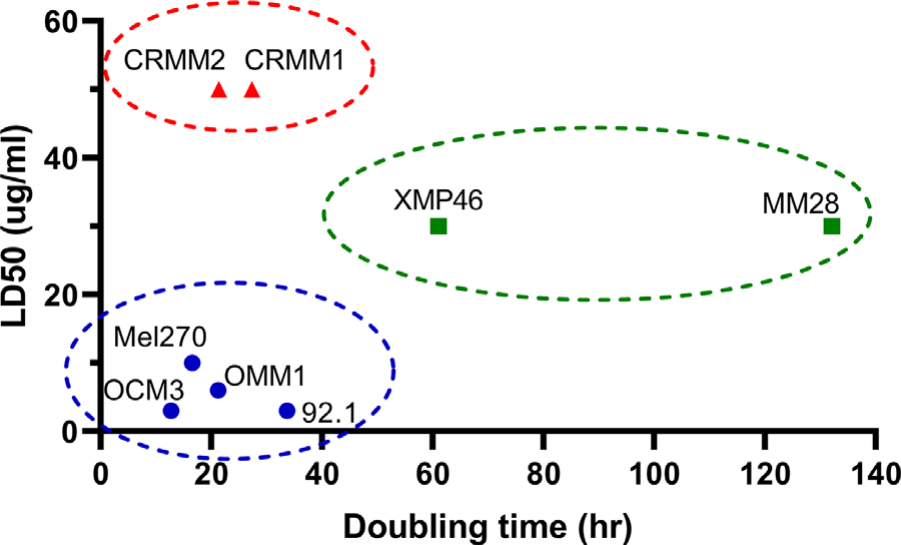
Doubling time and LD50 for each studied cell line. Cell growth doubling time was based on non-VP-treated cells in our specific experimental conditions. LD50 for VP was based on VP treatment at various dosages for each cell line. Values for CRMM1 and CRMM2 were arbitrarily cut off at a maximum of 50 ug/mL. Three clusters can be identified: **A** (*red*), CoM cell lines (*BRAF*/*NRAS*-mut). **B** (*green*), UM cell lines (BAP1-neg, *GNAQ*/*11*-mut). **C** (*blue*), UM cell lines (BAP1-pos, *GNAQ*/*11*-mut), and cutaneous melanoma cell line (*BRAF*-mut).

To examine the effect of growth rate on the susceptibility of cell lines to VP, we plotted the LD50 (as determined with the viability tests) against the doubling time (as determined with the cell growth experiments) of all cell lines. Three clusters of cells could be identified: (1) high doubling time and high LD50 (i.e. slow growing, insensitive to VP); (2) low doubling time and high LD50 (i.e. fast growing, insensitive to VP); and (3) low doubling time and low LD50 (i.e. fast growing, sensitive to VP). Cluster 1 comprises the BAP1-negative UM cell lines (XMP46 and MM28). Cluster 2 comprises the CoM cell lines (CRMM1 and CRMM2). Cluster 3 comprises the other, BAP1-positive, UM cell lines (92.1, MEL270, and OMM1) and melanoma cell line (OCM3; Fig. 4).

It can be deduced that, in order to be susceptible to VP, cell lines need a certain amount of cell growth, and a *GNAQ/11* mutation may lower the threshold for VP sensitivity.

### Protein Expression of YAP/TEAD and Downstream Actors CMYC/CYR61 Follows Cell Viability

To further understand the effects of VP on melanoma cells and the YAP/TAZ pathway in various cell lines, we performed Western Blot analyses of YAP, TEAD, and downstream target CMYC. Cell lines were cultured for 24 hours with a low dose liposomal VP in PBS (1.25 ug/mL), high dose liposomal VP in PBS (7.5 ug/mL), or control (PBS).

All BAP1-positive, *GNAQ/11*-mutant UM cell lines demonstrated a reduction of YAP, TEAD, and CMYC upon VP administration. This was similarly seen in cell line OCM3 (*BRAF*-mut) and to some extent in the *NRAS*-mutated cell line CRMM2. The rest of the cell lines (*BRAF*-mutated cell line CRMM1, and slow-growing BAP1-negative cell lines MM28 and XMP46) demonstrated little or no reduction of YAP, TEAD, or CMYC upon VP administration (Supplementary Fig. S1).

## Discussion

We observed that expression of several YAP/TAZ-related genes correlated with tumor genetics in UM, with a higher activity in M3/BAP1-negative lesions, although the prognostic value of the YAP/TAZ pathway was limited. Although most UM cell lines were sensitive to VP, two BAP1-negative UM cell lines, as well as two *BRAF/NRAS*-mutated CoM cell lines, were not. We found that not only the mutational background of the studied genes, but also cell growth rate was an important predictor of YAP/TAZ inhibition by VP, with a slow growth rate relating to VP insensitivity.

To our knowledge, we are the first to extensively relate the YAP1 pathway to genetic characteristics of UM, using a large set of patients with UM. When comparing mRNA expression of YAP1-related genes with clinical and genetic determinants, we found a higher expression level in UM with M3/BAP1-loss. The prognostic value of YAP1-related mRNA expression was limited, however, with only a high expression of *WWTR1* being significantly related to metastasis development. A recent study on mRNA data of the TCGA project on UM similarly identified no relation between *YAP1* gene expression and survival, but did not report on *WWTR1* or the relation with the genetic makeup of the tumors.^41^

Our experiments showed that exposure to VP decreased cell viability in BAP1-positive UM cell lines harboring mutations in *GNAQ/11*, as has been reported before.^35^^,^^57^ A mutation in *GNAQ/11* was no exclusive predictor of a response to VP, however, as we report on cell lines with a *GNAQ/11* mutation without a clear response (MM28 and MP46), and a cell line lacking *GNAQ/11* mutations that did demonstrate decreased survival (OCM3). We noticed that the nonresponding cell lines had a slower growth rate compared to the responding ones, and we hypothesize that this may have been limiting the susceptibility for YAP1-inhibition.

We expected that BAP1-negative UM cell lines would be more susceptible to YAP1-inhibition, because the YAP1 pathway was upregulated in BAP1-loss UM. Unexpectedly, these cell lines demonstrated very little response to VP; however, we noticed a remarkable slower growth rate compared to the BAP1-positive UM cell lines. An alternative explanation is that BAP1-loss results in YAP/TAZ pathway insensitivity, or that BAP1-loss causes a YAP1-independent growth disadvantage.

We also studied cell lines lacking a mutation in *GNAQ/11*. We identified no convincing effect of VP in the two CoM cell lines with either a *BRAF* or *NRAS* mutation (CRMM1 and CRMM2), whereas the cutaneous melanoma cell line OCM3 did show a response to VP. Notably, the growth rate of OCM3 was higher than that of CRMM1 and CRMM2. In line with our findings, previous work by Yu et al. showed a limited, yet present, response to VP for cell line OCM3, with about a halving of cell count compared to control after 3 days of treatment with high-dose VP.^35^ Our results may be more pronounced due to a longer, 6 day, treatment and addition of FBS to the cell culture medium (that is known to activate the Hippo pathway),^58^ but both studies confirm that cell lines lacking a *GNAQ/11*-mutation may be affected by VP.

As a treatment for UM, we concur with others reporting on the potential benefit of VP in preclinical models. Clinical experience shows that BAP1-mutated UM show more aggressive characteristics than BAP1-wildtype UM, however, which is opposite to the in vitro behavior of our cell line model with BAP1-loss. Unfortunately, no fast-growing UM cell line models lacking BAP1 are available. It would therefore still be interesting to test the susceptibility of BAP1-negative cells in vivo to VP treatment. It has been suggested that targeting the YAP1-pathway alone may not be the most effective route to attack UM, and that combined treatment aimed at the *GNAQ/11* pathway and other pathways such as *BAP1*,^59^ or at others^19^ would be more effective. Indeed, as VP only targets one arm of the G-coupled receptor network, it may be necessary to target multiple upstream nodal points to fully block the YAP1-pathway and it is likely that combinations of drugs are needed.^19^

Being the first to study VP in CoM, our results are not supportive for VP as a single-agent therapy in this disease. This may resemble earlier work on cutaneous melanoma cell lines that demonstrated mixed responses to YAP/TAZ inhibition: whereas reduced cell growth and reduced YAP/TAZ protein levels were reported after VP,^35^^,^^39^ inhibiting YAP/TAZ in cutaneous melanoma cell lines via shRNA, demonstrated no effect on proliferation in vitro.^40^ Similarly, whereas cutaneous melanoma xenograft mouse models demonstrated no tumor response to VP in one study,^39^ another study using shRNA inhibition of YAP/TAZ did identify a decreased in vitro invasiveness and less metastases formation in mice.^40^

A strength of our study is the availability of data on mRNA expression and genetic status of a large number of UM cases. We were also able to test a broad panel of cell lines, representing various mutational backgrounds of UM and CoM. Some conflicting findings were observed between mRNA expression of YAP-related genes in the TCGA data and Leiden data. This may be due to differences in the study group, as UM in the Leiden cohort were somewhat smaller than those analyzed in the TCGA project, which may have influenced YAP1 activity.

An interesting matter in cell line studies is whether cell lines mimic the traits of their original tumor type,^56^ and whether in vitro findings correspond to the in vivo situation. In our study, we find that YAP1-related genes are differentially expressed in UM tissue based on genetic traits (such as BAP1 loss). Because protein expression in our cell culture work was assessed using separate experiments, we cannot formally conclude on a differential baseline YAP1 expression between individual BAP1-positive and BAP1-negative cell lines. Importantly, all studied cell lines expressed YAP1 protein, allowing assessment of inhibition following VP treatment (see Supplementary Fig. S1), which was the aim of this study. The relevance of different baseline YAP1 expression levels between cell lines are difficult to assess, because external stimuli influence hippo-pathway activity,^38^ which is not modeled fully in vitro.

Interestingly, the YAP/TAZ pathway has recently been linked to mechanisms of resistance against targeted therapy and escape against immunotherapy in cancer.^60^^,^^61^ Upregulation of the YAP/TAZ pathway was found in cutaneous melanoma tissue of patients who developed resistance to BRAF-inhibitor or RAF + MEK-inhibitor therapy.^62^^,^^63^ Similar to these findings in cutaneous melanoma, upregulation of the YAP1 pathway was found in UM models after MEK-inhibition.^64^

Upregulation of the YAP/TAZ pathway has also been linked to several immune-suppressing effects, relevant for immunotherapy. YAP1 expression was positively associated with PD-L1 expression in samples of cutaneous melanoma, creating an escape for destruction by CD8+ T cells.^65^ Increased YAP1 was associated with lower expression of CD8, HLA class I molecules, and TAP1 in cutaneous melanoma tissue, similarly pointing toward decreased immune recognition.^62^

Blocking the YAP/TAZ pathway may be beneficial to overcome MAPK-inhibitor resistance, as YAP/TAZ knockdown restored sensitivity to BRAF-inhibitors in previously resistant cutaneous melanoma cell lines,^66^ and VP caused reduced tumor formation in a mouse model with BRAF-inhibitor-resistant skin melanoma cells.^67^ Even so, knockdown of YAP and TAZ caused reduced expression of PD-L1 in cutaneous melanoma cell lines,^65^ which would theoretically make these cells more vulnerable to CD8+ T cell attack.

The true future application of YAP/TAZ inhibition (as with VP) may therefore possibly not be as a single-agent therapy to any type of melanoma, but as an additive to other (targeted or immuno-) therapies. This would be beneficial in the treatment of UM as well as CoM, mirroring the findings from cutaneous melanoma.

Concluding, expression of YAP/TAZ-related genes correlated with tumor genetics in UM, with a higher activity in M3/BAP1-negative lesions. The prognostic value of YAP1-related gene expression on metastasis development was limited. Although most UM cell lines responded in vitro to VP, BAP1-negative UM cell lines and CoM cell lines did not. We find that not only the mutational background of the studied genes, but also cell growth rate is an important predictor of YAP/TAZ inhibition by VP. Our study implies a potential role for the YAP1 pathway as therapeutic target in UM, but finds a limited role for single-agent therapy in CoM. YAP1 inhibition may be used as a cotreatment with both targeted and immunotherapy, to overcome mechanisms of resistance and escape.

## Supplementary Material

Supplement 1

Supplement 2
