# Supplementary material for: Targeting the YAP/TAZ Pathway in Uveal and Conjunctival Melanoma With Verteporfin
Source: Invest Ophthalmol Vis Sci. 2021 Apr 2;62(4):3. doi: 10.1167/iovs.62.4.3 (PMC8024781; doi:10.1167/iovs.62.4.3)

Supplemental Figure 1. Protein expression of YAP1-related genes in studied cell lines following 24 hr incubation with verteporfin (low dose: 1.25 ug/ml, high dose: 7.5 ug/ml) or control (PBS). Coomassie blue staining was used to ensure equal loading.

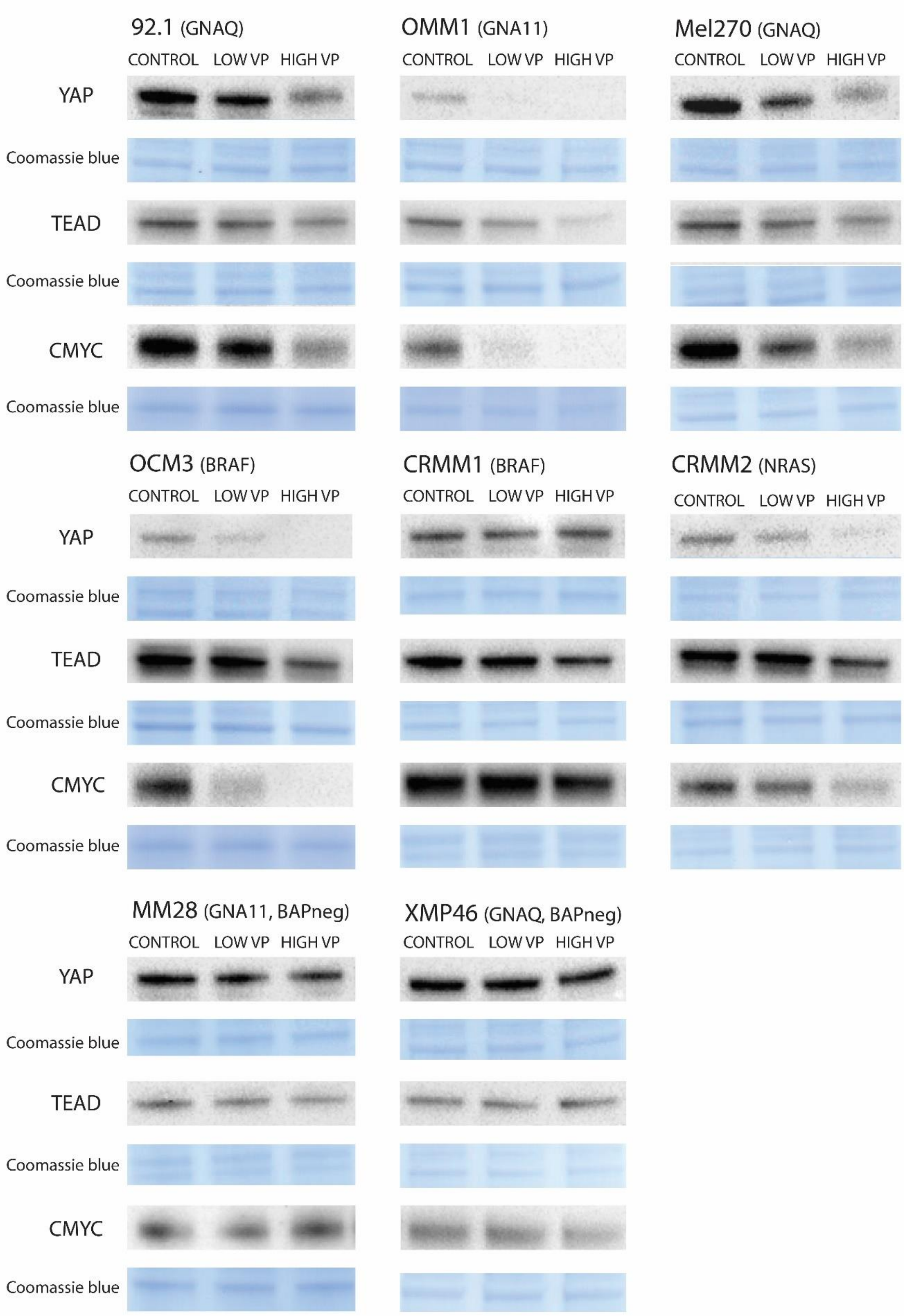

Supplement: Supplement 1 [file iovs-62-4-3_s001.pdf]
