# Supplementary material for: Targeting the YAP/TAZ Pathway in Uveal and Conjunctival Melanoma With Verteporfin
Source: Invest Ophthalmol Vis Sci. 2021 Apr 2;62(4):3. doi: 10.1167/iovs.62.4.3 (PMC8024781; doi:10.1167/iovs.62.4.3)
Supplement: Supplement 2 [file iovs-62-4-3_s002.pdf]

Supplemental Table 1. Characteristics of studied cell lines.

Cell line	92.1	Mel270	OMM1	XMP46	MM28	OCM3	CRMM1	CRMM2
Origin	UM	UM	UM	UM	UM	Skin Mel, [1]	Conj. Mel	Conj. Mel
Tissue	Primary	Primary	Metastasis	PDX, prim., [4]	PDX, met., [4]	Primary	Prim., [5]	Prim., [5]
Passage number	P23	P21	P6	P50	P40	P22	P75	P77
GNAQ	Q209L, [2]	Q209P, [2]	WT, [2]	c.626A>T, [4]	WT, [4]	WT, [2]	WT, [7]	WT, [7]
GNA11	WT, [2]	WT, [2]	Q209L, [2]	WT, [4]	c.626A>T, [4]	WT, [2]	WT, [7]	WT, [7]
BRAF	WT, [2]	WT, [2]	WT, [2]	ND	ND	V600E, [2]	V600E, [6]	WT, [6]
NRAS	WT, [2]	WT, [2]	WT, [2]	ND	ND	WT, [2]	WT, [6]	Q61L, [6]
BAP1 mutation	WT, [3]	WT, [3]		WT, [4]	c.1881C>A, [4]			
BAP1 IHC	Pos, [3]	Pos, [3]	Pos, [8]	Neg, [4]	Neg, [4]	Pos, [11]	ND, [7]	ND, [7]
Chr 3	Disomy, [3]	Disomy, [3]	ND, [7]	Monosomy, [4]	Monosomy, [4]	Disomy, [9] [10]	ND, [7]	ND, [7]
Chr 8	Gain 8q, [3]	Disomy 8q, [3]	ND, [7]	Gain 8q, [4]	Gain 8q, [4]		ND, [7]	ND, [7]
EIF1AX	C17G/A, [3]	WT, [3]	WT, [8]	WT, [4]	WT, [4]		ND, [7]	ND, [7]
SF3B1	WT, [3]	WT, [3]	WT, [8]	WT, [4]	WT, [4]		ND, [7]	ND, [7]
Original Reference	De Waard-Siebinga et al 1995 [50]	Verbik et al 1997 [52]	Luyten et al 1996 [51]	Amirouchene-Angelozzi et al 2014 [53]	Amirouchene-Angelozzi et al 2014 [53]	Huang et al 1994 [55]	Nareyeck et al 2005 [54]	Nareyeck et al 2005 [54]

Abbreviations: WT, Wild Type; ND, Not determined; Met, metastasis; Prim, primary; IHC, immunohistochemistry; Pos, positive; Neg, negative.

1. Cell line OCM3 was originally believed to be a UM cell line, but its origin was questioned[68] and it was found that it demonstrated many similarities with skin melanoma cell line SK-Mel28 [69].
2. Griewank et al [69]
3. Jager et al [56]
4. Amirouchene et al [53]
5. Nareyeck et al [54]
6. De Waard et al [70]
7. Bailey et al [71]
8. Bailey et al [71]
9. White et al [72]
10. Nareyeck et al [73]
11. Mosbeh et al [74]
12. Yu et al [35]
